# Supplementary material for: A biochemically-interpretable machine learning classifier for microbial GWAS
Source: Nat Commun. 2020 May 22;11:2580. doi: 10.1038/s41467-020-16310-9 (PMC7244534; doi:10.1038/s41467-020-16310-9)
Supplement: Supplementary file 2 — Supplementary Information [file 41467_2020_16310_MOESM2_ESM.pdf]

A biochemically-interpretable machine learning classifier for microbial GWAS

Kavvas et al.

SI Text, Figures, and Tables

Supplementary Figures **3**

Supplementary Figure 1: Example of metabolic connection between genetic variation and AMR in *Mycobacterium tuberculosis*. 3

Supplementary Figure 2: Overview of metabolic-model based sequence classifier for *M. tuberculosis* AMR 4

Supplementary Figure 3: Theory and detailed inference approach for Metabolic Allele Classifiers 5

Supplementary Figure 4: Bayesian Information Criteria distribution 6

Supplementary Figure 5: Flux GWAS Manhattan plots 7

Supplementary Figure 6: Ranking of significant alleles by conventional GWAS. 8

Supplementary Figure 7: Ranking of MAC objective coefficients. 9

Supplementary Tables **9**

Supplementary Table 1: 9

Supplementary Figures

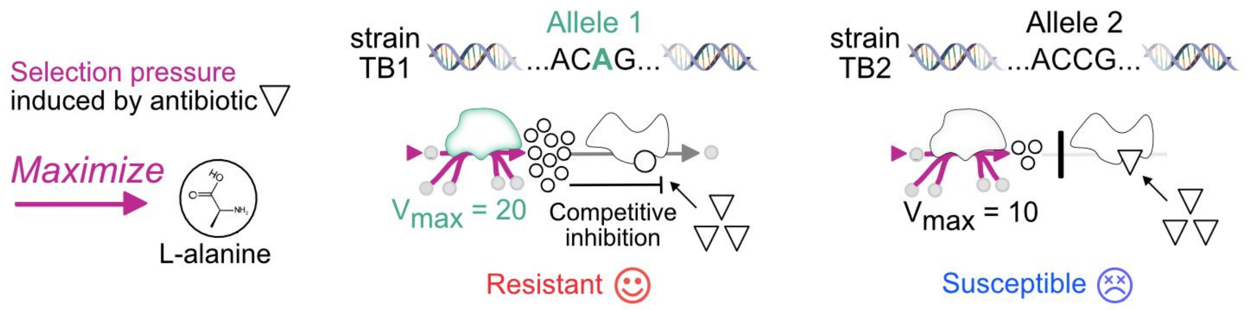

Supplementary Figure 1: Example of metabolic connection between genetic variation and AMR in *Mycobacterium tuberculosis*.

An allele (i.e., a unique variant of a gene) underlies resistance in strain TB1 by determining the maximum enzymatic flux of the protein it encodes. In this simple example, the antibiotic treatment causes a metabolic selection pressure to competitively inhibit antibiotic binding by maximizing synthesis of L-alanine, which is enabled by the allele-specific flux capacity. References for other similar and more complex metabolic examples are described in the text (**Supplementary Table 1**).

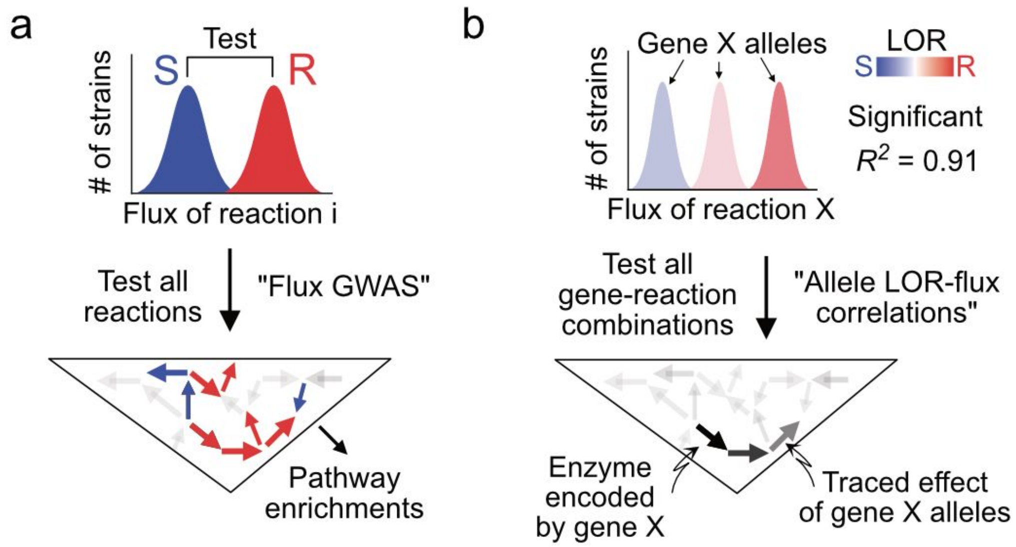

Supplementary Figure 2: Statistical analysis of fluxes distinguishing resistant and susceptible strains

(**a**) Statistical tests are performed on all strain-objective intersections to identify significant flux states discriminating between R and S strains (named "Flux GWAS"). (**b**) Statistical tests are also performed on the strain intersections to identify significant allele-specific fluxes and their network-level effects.

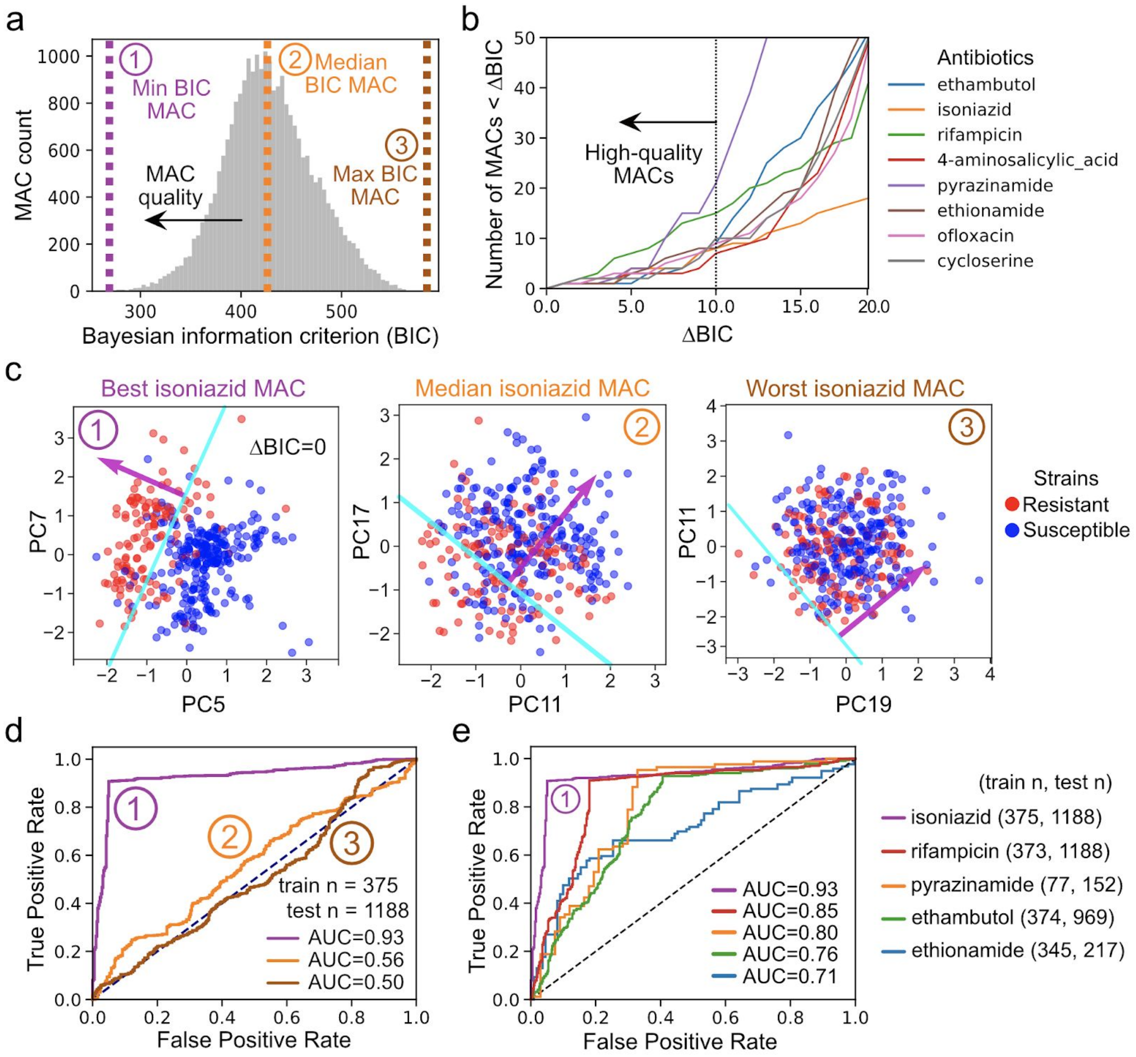

Supplementary Figure 3: Characteristics of estimated MACs

(a) Bayesian information criterion (BIC) distribution of 25,062 Metabolic Allele Classifiers (MACs) for isoniazid AMR. **(b)** Plot of the number of MACs that have less than a specific ΔBIC ($\Delta BIC_i = BIC_i - BIC_{min}$ for model *i*). Models with ΔBIC > 10 are generally considered to have insufficient support [31] **(c)** Plot of the two most significant principal component regression components (PC) for the minimum (left), median (middle), and maximum (right) isoniazid BIC MACs. The classification boundary (cyan) and MAC isoniazid-specific objective (purple) are shown. **(d)** Receiver operator characteristic (ROC) curves for the minimum, median, and maximum BIC isoniazid MACs determined using a test set of 1,188 isoniazid-tested strains. **(e)** Best MAC ROC curves for other antibiotics. Abbreviations: AUC, area under the curve.

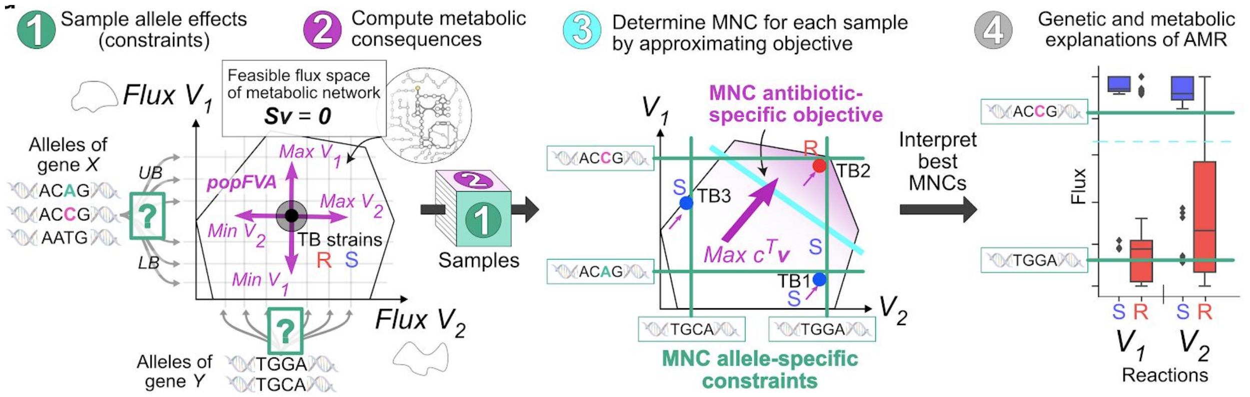

Supplementary Figure 4: Overview of metabolic-model based sequence classifier for *M. tuberculosis* AMR

The MAC is determined by three key steps: (1) generate an ensemble of random allele-constraint maps, (2) perform population flux variability analysis (popFVA) for each allele-constraint map, and (3) fit the popFVA solution with principal component logistic regression (PCR) to classify AMR phenotypes. The antibiotic-specific linear objective for each MNC sample is directly determined from the fitted antibiotic-specific PCR model, where popFVA variables v_{max} and v_{min} are replaced by MNC variables $v_{forward}$ and $v_{reversible}$, respectively (i.e., MNC objective is normal to the PCR decision boundary). The quality of the MNCs are then ranked with respect to each antibiotic using the bayesian information criterion (BIC).

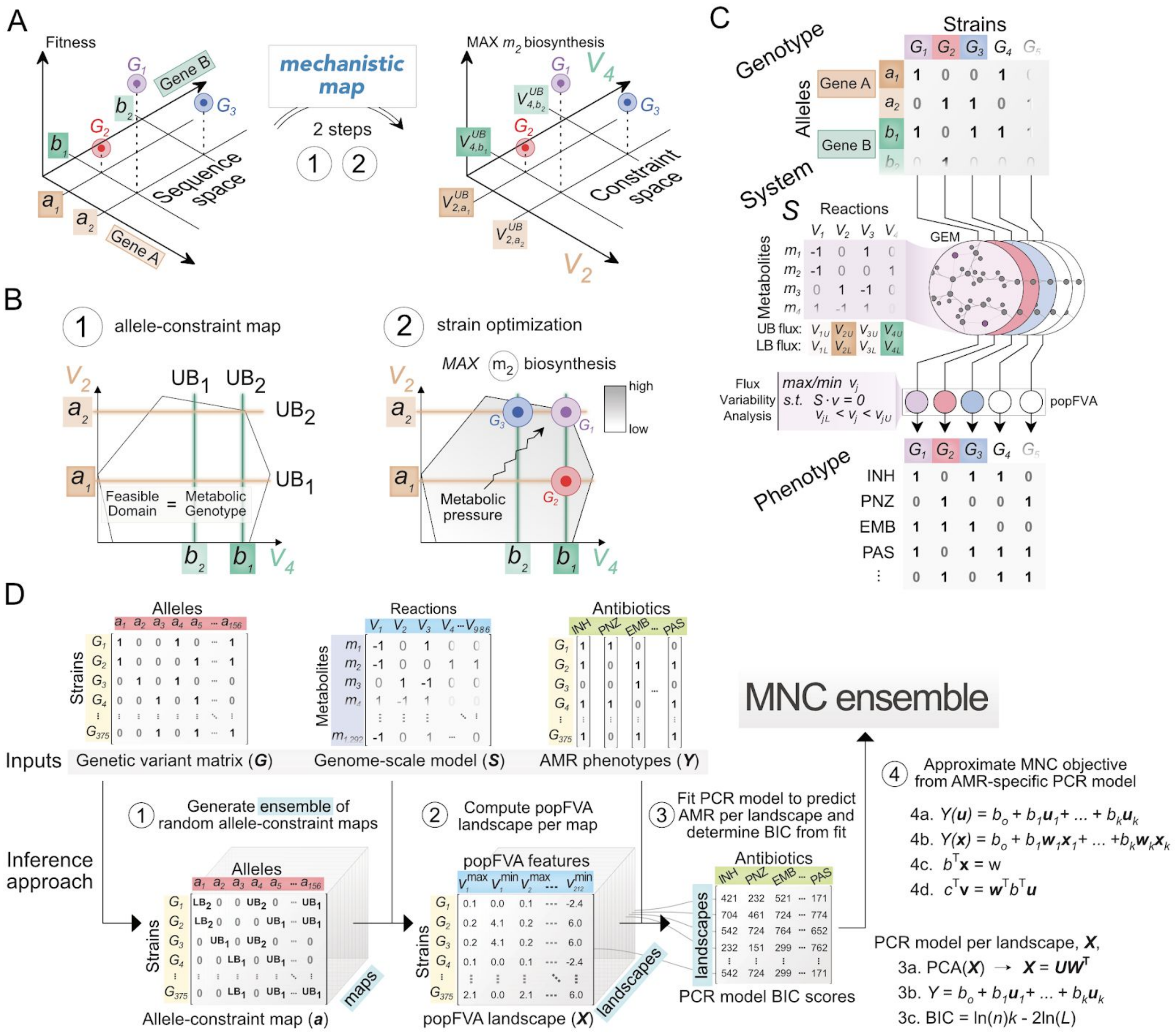

Supplementary Figure 5: Theory and detailed inference approach for Metabolic Allele Classifiers

(a) A fitness landscape illustration (left) for three strains (G_1, G_2, G_3), two genes (*A*, *B*), and two alleles per gene (a_1, a_2, b_1, b_2) is represented mechanistically (right) by two steps. **(b)** The mechanistic map involves two steps: (1) transforming sequencing space to constraint space by mapping each allele to a flux capacity constraint, and (2) strain-specific optimization of a biochemical objective. In this example, all alleles map to upper bound flux constraints and fitness is represented by metabolite m_2 biosynthetic capabilities. Our framework is thus an optimality approach to understanding evolutionary adaptation—in which an optimized objective is a proxy for fitness (i.e., AMR phenotype) and differential fitness results from differential constraints (Parker and Smith 1990). **(c)** Complete picture of mechanistic map between sequence variation and phenotypic variation. Flux variability analysis is computed for each strain-specific GEM to comprehensively evaluate the solution space resulting from the allele-specific constraints. We call this population level FVA, popFVA. **(d)** Inference of mechanistic maps through randomized sampling and model selection. Abbreviations: UB, upper bound; GEM; genome-scale model; GPR, gene-product-reaction rule.

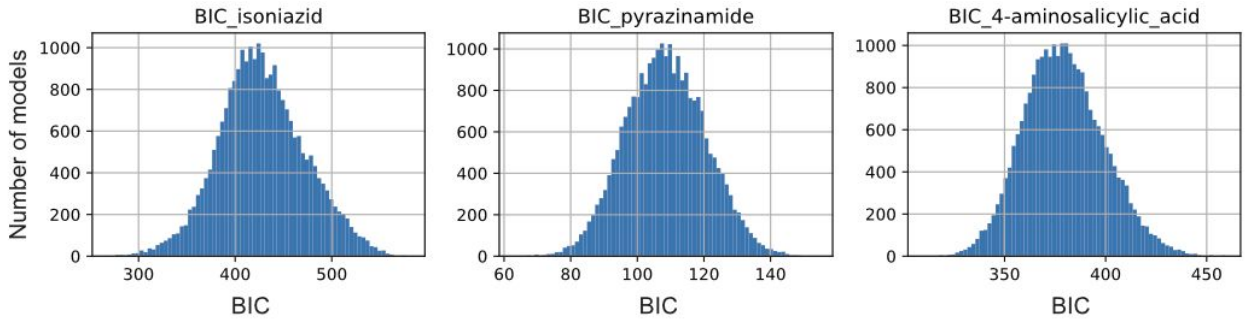

Supplementary Figure 6: Bayesian Information Criteria distribution

Bayesian information criteria (BIC) distribution of 25,062 metabolic network classifiers (MNCs) for isoniazid, pyrazinamide, and para-aminosalicylic acid AMR (left to right).

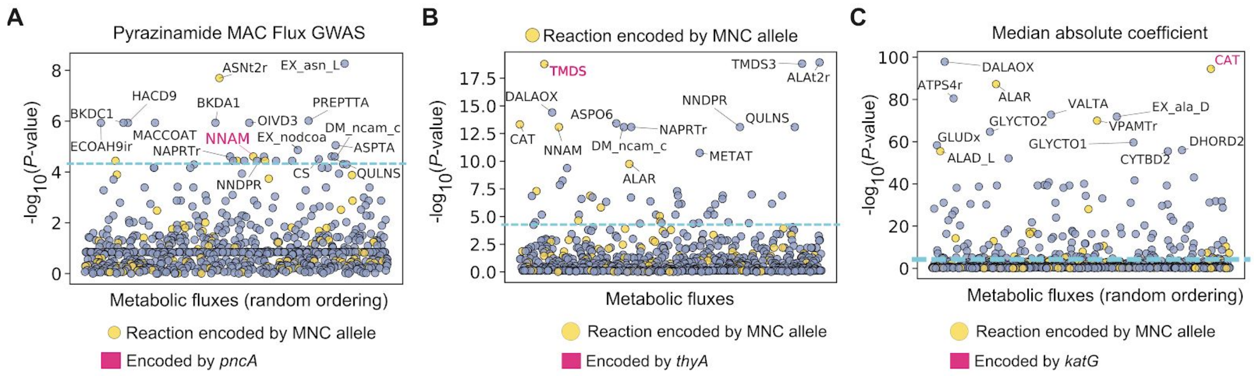

Supplementary Figure 7: Flux GWAS Manhattan plots

Manhattan plot of MAC reaction fluxes associated with (**A**) pyrazinamide AMR, (**B**) para-aminosalicylic acid AMR, and (**C**) isoniazid AMR. The blue line describes the significance threshold set by the Bonferroni correction ($P < 4.66\text{x}10^{-5}$). The x-axis is a randomized ordering of the reaction indices. The primary known genetic determinant for each antibiotic is noted in magenta.

Supplementary Tables

Supplementary Table 1:

Antibiotic	Accounted for in iEK1011	Not accounted for in iEK1011	Metabolic pressure or consequence
INH (8/12)	***katG*^, *inhA*^**, *fabG1, kasA, accD6, fadE24, fbpC,* ***ndh*,**	*oxyR-ahpC, iniABC*	V_{max} differences in *katG* and *inhA* AMR alleles (Quémard et al. 1995), Max NADH+/NAD ratio (Vilcheze et al. 2005)
RIF (0/2)	—	*rpoB*^, *rpoC*	—
EMB (3/4)	*embB*^, ***ubiA*^**, *aftA*	*embR*	Max DPA pool (Safi et al. 2013)
PNZ (1/1)	***pncA*^**, *ppsA*	—	*PZase* loss-of-function (Cheng et al. 2000), Max CoA depletion*, (Gopal et al. 2016; Rosen et al. 2017), Min PDIM biosynthesis* (Gopal et al. 2016),
OFX (0/2)	—	*gyrA*^, *gyrB*	—
PAS (3/3)	*folC*^, ***thyA*^, *ribD***	—	Max THF pool (Zheng et al. 2013), decreased *thyA* activity (V_{max}) (Rengarajan et al. 2004)
DCZ (3/3)	*ddl, alr*^, ***ald*,**	—	Max L-alanine pool (Desjardins et al. 2016)
STR (0/2)	—	*rpsL, gidB*	
ETA (2/2)	*ethA*^, ***mshA-D***	—	Min mycothiol biosynthesis (Vilchèze et al. 2008)
MDR and others	*dprE1, drrABC, moeW,*	***prpR***	Alteration of propionyl-CoA metabolism (Hicks et al. 2018),

^Primary AMR determinant

*Evidence for pyrazinoic acid (POA), the active form of PNZ

Supplementary Table 1. Evaluation of GEM scope in mechanistically describing variants of known *M. tuberculosis* AMR genes. Abbreviations: INH, isoniazid; RIF, rifampicin; EMB, ethambutol; PNZ, pyrazinamide; OFX, ofloxacin; PAS, para-aminosalicylic acid; ETA, ethionamide; STR, streptomycin; DCZ, D-cycloserine; DPA, decaprenylphosphoryl-b-D-arabinose; CoA, coenzyme A; PDIM, phthiocerol dimycocerosates; THF, tetrahydrofolate. Bolded genes correspond to the noted metabolic pressure or consequence.

Supplementary Notes

How alleles connect to flux constraints

Metabolic genes encode enzymes that catalyze metabolic reactions. Variation in the sequence of a gene (alleles) may result in variation in the 3D structure of the enzyme, which leads to variation in the catalytic function of the enzyme. The catalytic function of a metabolic enzyme is to transform metabolites (substrates) to other metabolites (products) at a rate known as metabolic flux (see Glossary). The rate of this transformation is known as metabolic flux. Therefore, the allele sequence determines the maximum and minimum flux of the enzymatic reaction it encodes for, which are described explicitly in flux balance analysis as constraints (V_{max}, V_{min} constraints). In a genome-scale model, genes are associated with reactions through the gene-protein-reaction (GPR) (see Glossary).

Computing the Metabolic Allele Classifiers

We utilized randomized sampling, machine learning, and model selection to identify predictive MACs (see **Supplementary Figures 2-3** and **Methods** for further details of the process outlined below). Specifically, we generated an ensemble of MAC models by randomly sampling allele-constraint maps, $\boldsymbol{a}^{lb,ub}$, and unbiasedly evaluating their metabolic consequences through population flux variability analysis (popFVA)—a population extension of FVA presented in this study [29]. For each sampled allele-constraint map, its corresponding popFVA result was fitted with L1-regularized principal component regression (PCR) to predict AMR phenotypes. The regression coefficients of the antibiotic-specific PCR models were then used to approximate antibiotic-specific objective functions for the MACs. The quality of each MAC in the ensemble

was then quantified using the Bayesian Information criterion (BIC) [30]—a model selection criterion that penalizes model complexity (i.e., number of parameters or "inefficiency").

The results provided here and throughout the main text correspond to an ensemble of 25,062 MACs, where each MAC was trained on the same set of 375 strains to predict antibiotic phenotypes. We limited the set of alleles modeled by the MAC to 237, describing 107 genes with known and implicated relations to AMR (**Supplementary File 1**). The allele-constraint map for each MAC was generated by sampling a uniform distribution of four constraints per allele (i.e., 2 lb, 2 ub), for all 237 alleles (see **Methods** for further discussion on determining the allele-specific constraint set by discretization of flux solution space).

Metabolic allele classifiers accurately and efficiently predict AMR

We find that the BIC of sampled MACs follows a normal distribution (**Fig. 2a,** see **Supplementary Figure 3**) where the number of high-quality MACs (i.e., $\Delta BIC_i<10$) per antibiotic ranges from 7 (cycloserine) to 21 (pyrazinamide) (**Fig. 2b**). The number of high-quality MACs reflects the parameter space, which is a function of both the training size and complexity of the fitted MAC. The majority of sampled MACs therefore lack sufficient empirical support according to the BIC metric [31], leaving only a select few MACs capable of downstream analysis.

Plotting the top two significant components of the PCR models corresponding to the minimum ("best"), median, and maximum BIC isoniazid MACs, showed that the best MAC explains AMR phenotypes with higher accuracy and less complexity (i.e., "prediction efficiency") than the other MACs (**Fig. 2c)**. High-quality MACs thus provide simple explanations for their predictions.

To validate the MACs and their relative quality, we used the minimum, median, and maximum BIC isoniazid MACs to predict AMR for a holdout test set of 1,182 isoniazid-tested TB strains and quantified their quality using the area under the receiver-operator characteristic curve (AUC). We find that the minimum BIC MAC achieves high performance (AUC=0.93) while the median (AUC=0.53) and maximum (AUC=0.50) BIC MACs perform poorly, which is consistent with our BIC-based assessment of MAC quality (**Fig. 2d)**. High-quality MAC predictions are therefore robust in accurately classifying strains not previously seen.

We assessed the best MACs for rifampicin, pyrazinamide, ethambutol, and ethionamide using held out test sets and find that the MACs generally achieve high classification performance (**Fig. 2e**), with scores similar to our previous mechanism-agnostic machine learning models [2]. These results show that the MAC performs on par with state-of-the-art machine learning approaches while maintaining low mechanistic model complexity.

Comparison between MAC and the Support Vector Machine

For the Support Vector Machine (SVM), the learned classifier has the following form,

$$H_{SVM} = \text{sign}(\mathbf{w}^T\mathbf{x}_k + b),$$

Where H>0 is resistant, H<0 is susceptible, and *x* describes the allele presence/absence vector of a particular strain. The parameters ***w*** and *b* are learned from the data through optimization.

For the MAC, the learned classifier has the following form,

$$H_{MAC} = \text{sign}(\max \mathbf{c}^T\mathbf{v} + b \text{ subject to } S\mathbf{v} = \mathbf{0},\ \mathbf{v}^{lb} \leq \mathbf{v} \leq \mathbf{v}^{ub},\ \mathbf{x}^T\mathbf{a}^{lb} < \mathbf{v} < \mathbf{x}^T\mathbf{a}^{ub}),$$

Where H>0 is resistant, H<0 is susceptible, ***x*** describes the allele presence/absence vector of a particular strain, ***v*** describes the flux state of the strain, S is the stoichiometric matrix, and ***a*** is

the mapping of alleles to lb and ub flux constraints. The parameters ***w***, *b*, and ***a*** are learned from the data through a detailed multi-step optimization process (see **Methods**).

Design choices for the MAC

The outcome of the MAC depends on two major design choices: the set of alleles and the objective function that optimally separates strains into resistant and sensitive strain cohorts in the overall metabolic flux space. Although our approach does not explicitly require prior knowledge of key AMR genes, we chose a set of alleles with just over 100 genes with known and implicated AMR relations in order to both provide test cases and to address the combinatorial explosion of sampling possible allelic effects. Relaxing the current computational bottleneck in identifying MACs will enable the utilization of all alleles. For determining the objective function, our approach was based on the key insight that a linear program may behave as a machine learning classifier if its objective optimizes in the direction normal to a predictive classification plane. While we utilized PCA, L1-logistic regression, and the BIC metric to identify sparse linear objectives, there are potentially alternative avenues that could be taken. The major concept that should sustain in any model selection strategy is that a good model is simple (in structure) yet accurate (in its predictions). Application of the MAC to other GWAS datasets may therefore benefit from tuning these parameters appropriately.

Glossary

Metabolic Reaction: The transformation of reactant metabolites to product metabolites. Most metabolic reactions are enabled by proteins called enzymes.

Protein-coding sequence (CDS): A particular sequence on the genome that encodes a protein.

Metabolic Flux: The rate of a metabolic reaction.

Metabolic Pathway: A human-defined set of metabolic reactions.

Gene-Product-Reaction (GPR): The association between genes and reactions described in a genome-scale model.

References

Cheng, S. J., L. Thibert, T. Sanchez, L. Heifets, and Y. Zhang. 2000. "pncA Mutations as a Major Mechanism of Pyrazinamide Resistance in Mycobacterium Tuberculosis: Spread of a Monoresistant Strain in Quebec, Canada." *Antimicrobial Agents and Chemotherapy* 44 (3): 528–32.

Desjardins, Christopher A., Keira A. Cohen, Vanisha Munsamy, Thomas Abeel, Kashmeel Maharaj, Bruce J. Walker, Terrance P. Shea, et al. 2016. "Genomic and Functional Analyses of Mycobacterium Tuberculosis Strains Implicate Ald in D-Cycloserine Resistance." *Nature Genetics* 48 (5): 544–51.

Gopal, Pooja, Michelle Yee, Jickky Sarathy, Jian Liang Low, Jansy P. Sarathy, Firat Kaya, Véronique Dartois, Martin Gengenbacher, and Thomas Dick. 2016. "Pyrazinamide Resistance Is Caused by Two Distinct Mechanisms: Prevention of Coenzyme A Depletion and Loss of Virulence Factor Synthesis." *ACS Infectious Diseases* 2 (9): 616–26.

Hicks, Nathan D., Jian Yang, Xiaobing Zhang, Bing Zhao, Yonatan H. Grad, Liguo Liu, Xichao Ou, et al. 2018. "Clinically Prevalent Mutations in Mycobacterium Tuberculosis Alter Propionate Metabolism and Mediate Multidrug Tolerance." *Nature Microbiology* 3 (9): 1032–42.

Parker, G. A., and J. Maynard Smith. 1990. "Optimality Theory in Evolutionary Biology." *Nature* 348 (November): 27.

Quémard, A., J. C. Sacchettini, A. Dessen, C. Vilcheze, R. Bittman, W. R. Jacobs Jr, and J. S. Blanchard. 1995. "Enzymatic Characterization of the Target for Isoniazid in Mycobacterium Tuberculosis." *Biochemistry* 34 (26): 8235–41.

Rengarajan, Jyothi, Christopher M. Sassetti, Vera Naroditskaya, Alexander Sloutsky, Barry R. Bloom, and Eric J. Rubin. 2004. "The Folate Pathway Is a Target for Resistance to the Drug Para-Aminosalicylic Acid (PAS) in Mycobacteria." *Molecular Microbiology* 53 (1): 275–82.

Rosen, Brandon C., Nicholas A. Dillon, Nicholas D. Peterson, Yusuke Minato, and Anthony D. Baughn. 2017. "Long-Chain Fatty Acyl Coenzyme A Ligase FadD2 Mediates Intrinsic Pyrazinamide Resistance in Mycobacterium Tuberculosis." *Antimicrobial Agents and Chemotherapy* 61 (2). https://doi.org/10.1128/AAC.02130-16.

Safi, Hassan, Subramanya Lingaraju, Anita Amin, Soyeon Kim, Marcus Jones, Michael Holmes,

Michael McNeil, et al. 2013. “Evolution of High-Level Ethambutol-Resistant Tuberculosis through Interacting Mutations in Decaprenylphosphoryl-[beta]-D-Arabinose Biosynthetic and Utilization Pathway Genes.” *Nature Genetics* 45 (10): 1190–97.

Vilchèze, Catherine, Yossef Av-Gay, Rodgoun Attarian, Zhen Liu, Manzour H. Hazbón, Roberto Colangeli, Bing Chen, et al. 2008. “Mycothiol Biosynthesis Is Essential for Ethionamide Susceptibility in Mycobacterium Tuberculosis.” *Molecular Microbiology* 69 (5): 1316–29.

Vilcheze, Catherine, Torin R. Weisbrod, Bing Chen, Laurent Kremer, Manzour H. Hazbón, Feng Wang, David Alland, James C. Sacchettini, and William R. Jacobs. 2005. “Altered NADH/NAD+ Ratio Mediates Coresistance to Isoniazid and Ethionamide in Mycobacteria.” *Antimicrobial Agents and Chemotherapy* 49 (2): 708–20.

Zheng, Jun, Eric J. Rubin, Pablo Bifani, Vanessa Mathys, Vivian Lim, Melvin Au, Jichan Jang, et al. 2013. “Para-Aminosalicylic Acid Is a Prodrug Targeting Dihydrofolate Reductase in Mycobacterium Tuberculosis.” *The Journal of Biological Chemistry* 288 (32): 23447–56.
