# Supplementary material for: A biochemically-interpretable machine learning classifier for microbial GWAS
Source: Nat Commun. 2020 May 22;11:2580. doi: 10.1038/s41467-020-16310-9 (PMC7244534; doi:10.1038/s41467-020-16310-9)
Supplement: Supplementary file 9 — Reporting Summary [file 41467_2020_16310_MOESM9_ESM.pdf]

nature research

Corresponding author(s): Bernhard Palsson

Last updated by author(s): April 6, 2020

Reporting Summary

Nature Research wishes to improve the reproducibility of the work that we publish. This form provides structure for consistency and transparency in reporting. For further information on Nature Research policies, see Authors & Referees and the Editorial Policy Checklist .

Statistics

For all statistical analyses, confirm that the following items are present in the figure legend, table legend, main text, or Methods section.

n/a	Confirmed	
☐	☒	The exact sample size (*n*) for each experimental group/condition, given as a discrete number and unit of measurement
☐	☒	A statement on whether measurements were taken from distinct samples or whether the same sample was measured repeatedly
☐	☒	The statistical test(s) used AND whether they are one- or two-sided<br>*Only common tests should be described solely by name; describe more complex techniques in the Methods section.*
☒	☐	A description of all covariates tested
☐	☒	A description of any assumptions or corrections, such as tests of normality and adjustment for multiple comparisons
☒	☐	A full description of the statistical parameters including central tendency (e.g. means) or other basic estimates (e.g. regression coefficient) AND variation (e.g. standard deviation) or associated estimates of uncertainty (e.g. confidence intervals)
☒	☐	For null hypothesis testing, the test statistic (e.g. *F*, *t*, *r*) with confidence intervals, effect sizes, degrees of freedom and *P* value noted<br>*Give P values as exact values whenever suitable.*
☒	☐	For Bayesian analysis, information on the choice of priors and Markov chain Monte Carlo settings
☒	☐	For hierarchical and complex designs, identification of the appropriate level for tests and full reporting of outcomes
☐	☒	Estimates of effect sizes (e.g. Cohen's *d*, Pearson's *r*), indicating how they were calculated

Our web collection on statistics for biologists contains articles on many of the points above.

Software and code

Policy information about availability of computer code

Data collection	No software was used to collect data utilized in this study.
Data analysis	Descriptions of the open source and custom code utilized in this study are provided at the following github repository (https://github.com/erolkavvas/metabolic-allele-classifiers).

For manuscripts utilizing custom algorithms or software that are central to the research but not yet described in published literature, software must be made available to editors/reviewers. We strongly encourage code deposition in a community repository (e.g. GitHub). See the Nature Research guidelines for submitting code & software for further information.

Data

Policy information about availability of data

All manuscripts must include a data availability statement. This statement should provide the following information, where applicable:

- Accession codes, unique identifiers, or web links for publicly available datasets
- A list of figures that have associated raw data
- A description of any restrictions on data availability

The TB AMR datasets utilized in this study were acquired from a previous study that performed machine learning and protein structure analysis. References describing this data set are provided in the supplementary information of the previous study 2. The dataset was initially acquired from the PATRIC database 26. The sequencing and phenotypic testing data for these strains were generated at the Broad Institute. Additional information for these sequencing projects can be found at the Broad Institute website for the TB Antibiotic Resistance Catalog (TB-ARC).

Field-specific reporting

Please select the one below that is the best fit for your research. If you are not sure, read the appropriate sections before making your selection.

- [x] Life sciences
- [ ] Behavioural & social sciences
- [ ] Ecological, evolutionary & environmental sciences

For a reference copy of the document with all sections, see nature.com/documents/nr-reporting-summary-flat.pdf

Life sciences study design

All studies must disclose on these points even when the disclosure is negative.

Sample size	No sample size calculation was performed. We chose 1,595 strains that had varying amounts of available antibiotic resistance phenotypes. These 1,595 strains were previously analyzed using machine learning to identify genetic determinants, and are therefore an ideal candidate for our mechanism-based machine learning approach.
Data exclusions	All the acquired strain sequences were utilized in this study.
Replication	No measurements were taken in this study.
Randomization	The total set of samples were randomly split into training size of 375 and test size of 1220. The random split was performed using the function "train_test_split" in scikit learn version 0.21.2.
Blinding	Blinding was not relevant to our study.

Reporting for specific materials, systems and methods

We require information from authors about some types of materials, experimental systems and methods used in many studies. Here, indicate whether each material, system or method listed is relevant to your study. If you are not sure if a list item applies to your research, read the appropriate section before selecting a response.

Materials & experimental systems

n/a	Involved in the study	
[x]	[ ]	Antibodies
[x]	[ ]	Eukaryotic cell lines
[x]	[ ]	Palaeontology
[x]	[ ]	Animals and other organisms
[x]	[ ]	Human research participants
[x]	[ ]	Clinical data

Methods

n/a	Involved in the study	
[x]	[ ]	ChIP-seq
[x]	[ ]	Flow cytometry
[x]	[ ]	MRI-based neuroimaging
