# Supplementary material for: A biochemically-interpretable machine learning classifier for microbial GWAS
Source: Nat Commun. 2020 May 22;11:2580. doi: 10.1038/s41467-020-16310-9 (PMC7244534; doi:10.1038/s41467-020-16310-9)
Supplement: Supplementary file 10 — Description of Additional Supplementary Files [file 41467_2020_16310_MOESM10_ESM.pdf]

Title: Supplementary Data File 1
Description: Excel sheets contained curated information for TB AMR genes and alleles.

Title: Supplementary Data File 2
Description: Dataframe for curated gene-pathway annotations.

Title: Supplementary Data File 3
Description: Excel sheets for pyrazinamide MAC characteristics.

Title: Supplementary Data File 4
Description: Excel sheets for 4-aminosalicylic acid MAC characteristics.

Title: Supplementary Data File 5
Description: Excel sheets for isoniazid MAC characteristics.

Title: Supplementary Data File 6
Description: Conventional results. Excel sheets describing bonferroni-corrected allele-AMR ANOVA F-tests and FDR-corrected pathway enrichments for isoniazid, pyrazinamide, and 4-aminosalicylic acid.
